# Labeling Nodes Using Three Degrees of Propagation

**DOI:** 10.1371/journal.pone.0051947

**Published:** 2012-12-28

**Authors:** Sara Mostafavi, Anna Goldenberg, Quaid Morris

**Affiliations:** 1 Department of Computer Science, Stanford University, Palo Alto, California, United States of America; 2 Sick Kids Research Institute, and Department of Computer Science, University of Toronto, Toronto, Canada; 3 Department of Molecular Genetics, Department of Computer Science, and the Donnelly Centre, University of Toronto, Toronto, Canada; King Abdullah University of Science and Technology, Saudi Arabia

## Abstract

The properties (or labels) of nodes in networks can often be predicted based on their proximity and their connections to other labeled nodes. So-called “label propagation algorithms” predict the labels of unlabeled nodes by propagating information about local label density iteratively through the network. These algorithms are fast, simple and scale to large networks but nonetheless regularly perform better than slower and much more complex algorithms on benchmark problems. We show here, however, that these algorithms have an intrinsic limitation that prevents them from adapting to some common patterns of network node labeling; we introduce a new algorithm, 3Prop, that retains all their advantages but is much more adaptive. As we show, 3Prop performs very well on node labeling problems ill-suited to label propagation, including predicting gene function in protein and genetic interaction networks and gender in friendship networks, and also performs slightly better on problems already well-suited to label propagation such as labeling blogs and patents based on their citation networks. 3Prop gains its adaptability by assigning separate weights to label information from different steps of the propagation. Surprisingly, we found that for many networks, the third iteration of label propagation receives a negative weight.

**Availability:**

The code is available from the authors by request.

## Introduction

In protein interaction networks, proteins linked by short paths of interactions tend to have similar functions [1], so an uncharacterized protein’s local network neighborhood can be used to predict its function [2]. Similarly in social networks, people connected by up to three degrees of separation can be used to predict one another’s happiness [3], risk of obesity [4], and which products they will purchase [5], [6]. Algorithms that predict node properties based on network connectivity are also important in web search [7], [8] and finding genes associated with genetic diseases [9].

These algorithms take as input a network that represents a set of objects as nodes whose pairwise relationships are encoded as the links in the network. Then, based on a query list of nodes with a particular property (or label) of interest, *i.e.,* “positive examples” or simply “positives”, these algorithms assign a score to each node in the network according to how likely it is to also have the queried label. For example, given a set of proteins with known functions in mitochondrial biogenesis, these algorithms can use the network to find other proteins likely to have the same function (*e.g.,* [10]–[12]); or given a set of people who have bought a particular product or service, these algorithms can find others likely to buy that product (*e.g.,* [6]).

Labeling problems like these have proved difficult when the network has a relatively large number of nodes compared to the number of positive examples, especially when the number of positive examples is small; to date the best performing algorithms for these problems, so-called label propagation algorithms [13]–[15], only perform well when linked nodes tend to share the same label (in other words, are assortatively mixed [16]). Label propagation fails for other, common patterns of node labeling [17], [18] such as disassortative mixing where linked nodes tend to have different labels (*e.g.,* people linked by sexual contact tend to be of different genders) or networks where nodes with many shared neighbors are more likely to have the same label than nodes that are directly connected to one another (*e.g.,* networks of negative genetic interactions [19], [20]).

Here we introduce a unifying framework that generalizes a large class of algorithms for node label prediction. We will refer to this general framework as Generic Label Propagation (GLP). This framework allows us to highlight a limiting underlying assumption shared by all algorithms that fall under this class. Further, using this framework, we introduce a new algorithm called 3Prop that retains all of the advantages of label propagation but can adapt to diverse node labeling patterns. In particular, 3Prop gains this adaptivity by learning independent weights for the first three steps of label propagation, thereby overcoming an inherent limitation that we show restricts other label propagation algorithms. 3Prop can be applied to large networks, computes node scores quickly, and is easy to implement. Furthermore, as we will show, because the topological structure of many real world networks limits the amount of node label information available through label propagation, 3Prop will likely perform well on a wide range of network labeling problems. Specifically, 3Prop predicts node labels more accurately (and sometimes much more accurately) than label propagation on five separate social and biological network labeling problems where the networks range in size from 750 to 3 million nodes and the proportion of labeled nodes ranges from 0.0002% to 40%.

## Methods

To motivate our 3Prop algorithm and to illustrate why label propagation fails for some networks, we introduce a general framework, which we will refer to as Generic Label Propagation (GLP), that encompasses common variations of label propagation algorithms. As we show, these algorithms fall into one of two classes, which we call *symmetric* and *asymmetric*. In both classes, the scores assigned to a node can be derived from probabilities that, starting from the node, random walks of different lengths will end in a positive node. These two classes have direct correspondence, in that algorithms from one class can be used to calculate scores for the other, and vice versa.

Below, we will first establish the GLP framework, and then use the random walk interpretation of the GLP scores to illustrate the intrinsic limitation of algorithms that fall into this framework.

### Generic Label Propagation

Label propagation algorithms address the following problem: given an undirected, possibly weighted, network over *n* nodes and a set of positive examples of nodes with the label of interest (*i.e.,* positives) as input, score all nodes in the network so as to rank them according to how likely they are to be positives. Note that in our formulation, we are not using negative examples, as in most problems that we consider (*e.g.,* predicting gene function) negative labels are rarely available. We will represent the input network using an 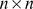 affinity matrix *A*, where 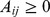 is the weight of the link between node *i* and *j* (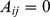 indicates that *i* and *j* are not connected). We will also assume that the network has no self-links so 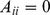 for all *i*. We represent the positive nodes using a label vector 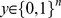, where 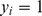 if node *i* is labeled (a positive) and 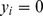 otherwise. For unweighted networks 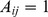 if nodes *i* and *j* are connected, in this case *A* is an adjacency matrix. In many real-world networks, only a small proportion of the node pairs are linked, making *A* a sparse matrix.

Label propagation assigns scores to nodes by an iterative process which propagates “evidence for positiveness” out from positive nodes through the links in the network to nearby nodes; this process is often compared to heat diffusion [13], [19]. On appropriately normalized networks, this iterative process is guaranteed to converge, and can be implemented using a simple update rule that can either be iterated to convergence [13], [14] or a fixed number of iterations [21]. The solution to this iterative process can also be derived by optimizing an objective function which corresponds to doing Maximum A Priori (MAP) estimation in the framework of Gaussian Markov Random Fields [13] (see Text S1), however we describe GLP through this iterative process.

In particular, in each iteration of GLP, the score of node *i*, given by 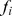, is updated by taking a weighted sum of the scores of *i*’s neighbors at the previous iteration, along with *i*’s initial label. Typically, to ensure convergence of the updates, the original matrix *A* is normalized to generate a matrix *M* that has the same pattern of non-zero elements and, therefore, corresponds to a network with the same links but different link weights. These normalizations are described later. Using *M*, the update rule for node *i* is given by:
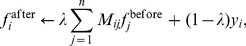
(1)where 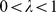 is a parameter that determines the influence of a node’s neighbors relative to its provided label. The update rule can be written in matrix-vector notation as 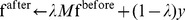; and, by expanding the iterations, the values of the node scores after *R* iterations, 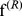, can be written as:
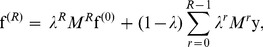
(2)where 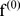 is the vector of the initial node scores and 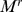 is the r-th matrix power of M. In the limit as 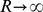, this series is guaranteed to converge to a unique solution so long as all the eigenvalues of M are in the range 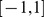. The final node scores at convergence, f, do not depend on 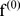, so, abusing notation, we can write:



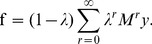
(3)Although this is the form we will consider in this paper, GLP algorithms typically compute f by rewriting the fixed-point equation corresponding to the update rule in equation (2), *i.e.,*

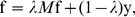
(4) as 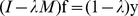 (where *I* is the identity matrix) and then either solving a linear system with coefficient matrix 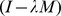 using a conjugate-gradient based algorithm [22], [23].

### Normalization and Two Variants of GLP

Two different normalizations of *A* ensure convergence and correspond to the asymmetric and symmetric variants of GLP (abbreviated here as ALP and SLP, respectively).

In SLP, the matrix 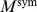 is produced by setting 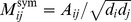, where 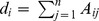 is the weighted degree of node *i*. In matrix notation, we can write 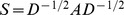 where we are using *S* to refer to 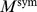 and *D* is a diagonal matrix whose diagonal elements 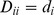. SLP methods include diffusion kernel-based [19] and Gaussian smoothing methods [13]. Other related approaches include the Iterated Laplacian method [24] and various methods derived by enforcing smoothness over a symmetric, positive-semi definite, kernel [25] (see Text S1).

In ALP, 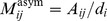, *i.e.,* 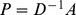 where *P* refers to 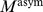. Note that unlike *S*, *P* is not symmetric. However, each row of *P* can be interpreted as a probability distribution over the neighbors of the corresponding node, *i.e., P* is a singly stochastic matrix. ALP methods include random walk with restart [26], personalized PageRank [7], and RankProp [21].

The solutions of SLP and ALP are closely related–a slightly modified version of the former can be used to compute node scores for the latter (and vice versa). This similarity arises because 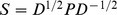, so 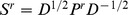. Substituting this definition into equation 3, we can write the final node scores for SLP, 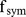, as:
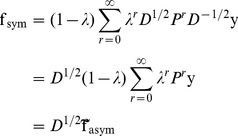
(5)where 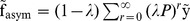 are node scores calculated by a slightly modified version of ALP that replaces y with a modified vector 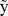 where 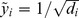 if node *i* is positive and 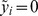 otherwise. The only differences between the two variants of GLP are an element-wise rescaling of the label vector and of the final node scores, so ALP can be used to compute the SLP node scores and vice versa. Although in our experimental section we use SLP because it performs slightly better than ALP on the labeling problems we consider, we will analyze ALP because the elements of matrix powers of *P* can be interpreted as random walk probabilities.

### Random Walk Interpretation of Label Propagation Scores and Inherent Limitations of GLP

If we interpret 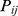 as the probability that a random walk of length one that starts from node *i* ends in node *j*, then the 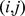-element of 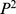, 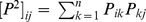, is the probability that a random walk of length two starting from node *i* will end in node *j*, and by induction, 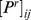 is the probability that a length *r* random walk starting from node *i* ends in node *j*. Under this interpretation, if we write 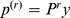 for the result of the matrix-vector product in the *r*-th term in the summation in equation 3, then its *i*-th element 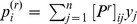 is the probability that a random walk of length *r* from node *i* will end in a positive node (recall that 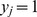 if node *j* is a positive and 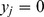 otherwise). As a result, the score assigned to node *i* by ALP, 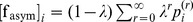, is a weighted sum of these random walk probabilities, where 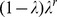 is the weight assigned to the length *r* random walk probabilities.

**Figure 1:**
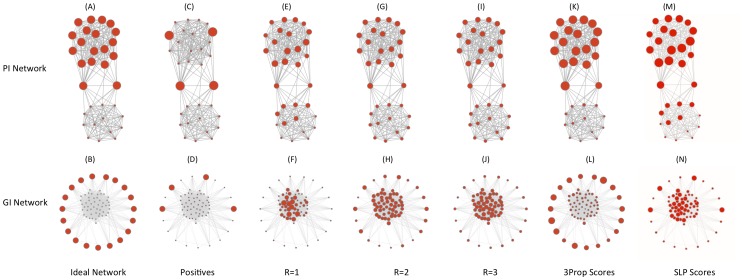
Node prediction scores assigned by 3Prop on two different types of network. Displayed networks are subnetworks of a protein interaction network (top row) and a genetic interaction network (bottom row). Both networks are derived from the BioGRID database, and the true positive examples are derived from Gene Ontology. (A): Large red nodes indicate proteins involved in meiotic cell cycle, (B): Large red nodes indicate proteins involved in transcription initiation. (C,D): Large red nodes indicate four randomly selected positives selected from (A) and (B) respectively, for training 3Prop. (E-J): Node size reflects relative magnitude of scaled random walk probabilities, 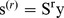, for 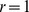 (E,F), 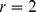 (G,H), or 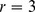 (I,J). (K,L): node scores assigned by 3Prop, compiled as a weighted sum of the three sets of the scaled random walk probabilities. (M,N): node scores assigned by GLP, compiled as an exponentially decaying weighted sum of the three random walk probabilities (we set 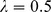 based on cross-validation).

Because 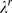
’s (weights) are always positive, a path of 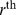 length will always have some input into the score, regardless of whether its relevant or not. Moreover, due to exponential decaying weights, direct neighbors will always have more influence on the score than second degree neighbors, and so on. This setup makes it impossible for label propagation to do well in cases such as dissasortative mixing [16] where direct links between nodes provide evidence against them having the same label (see Figure 1). In particular, in such scenario, 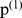 should have a negative weight to decrease the scores of nodes directly connected to positives. Similarly, when nodes with the same label tend to share neighbors, length two random walk probabilities should have a higher weight than direct connections but this is impossible under GLP because 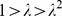. Note that the relative weight of 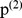 cannot be increased by setting 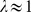 (so that 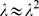) because this also assigns high weight to random walks of length 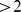 which degrades the quality of the node scores. Indeed, as we will show, versions of GLP with high values of 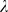 but with the summation in equation 3 truncated at 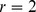 or 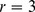 achieve higher accuracies on biological node labeling problems than GLP.

### 3Prop

The 3Prop algorithm makes GLP more adaptive by assigning independent weights to each of the first three summands (corresponding to random walks of up to length three) in equation 3. The number of free parameters is kept small in 3Prop because longer random walks are assigned zero weights. As we explain later, in many real-world networks, assigning non-zero weights to longer random walks is unnecessary and often counter-productive (see Subsection “Why Random Walks of Length Three?” in results). Another difference from GLP is that some of the weights can be negative, allowing 3Prop to adapt to disassortative mixing. Specifically, the 3Prop scores are given by:
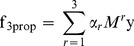
(6)for any real-valued scalars 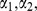 and 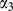. Note that because the scale of the node scores is arbitrary, there are fewer than three free parameters in 3Prop. Like GLP, there are two versions of 3Prop, symmetric and asymmetric, and the symmetric version of 3Prop (where 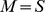) performs better than the asymmetric version (where 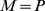) on the labeling problems we consider in the experimental section.

### Estimating 3Prop Weights

3Prop uses linear discriminant analysis (LDA) (see, *e.g.,* [27] for a description) to fit its weights to a given labeling problem. LDA is a linear classification algorithm that computes the 3Prop weights 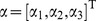 by maximizing the difference between the average 3Prop score of all positive and all non-positive nodes, while accounting for the correlation between random walks of various lengths. The value of 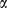 computed by LDA is given by:
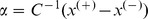
where 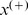 and 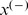 are vectors containing three elements, with the *r*-th element equal to the average score assigned to the positive and non-positive examples, respectively, when only considering random walks of length *r*. Specifically, in asymmetric 3Prop, 
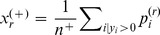
 and 
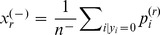
 where 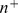 and 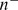 are the number of positive and non-positive examples, respectively (note that 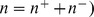. Recall that 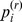 is the *i*-th element of the vector 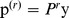. *C* is the sample covariance matrix of 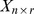 where 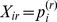. In the symmetric version of 3Prop, 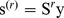 replaces 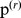. To avoid over-fitting, we compute 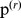
’s (or 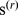
’s) on a randomly selected portion of the training data (generally the labels of 2/3 of the nodes), and then compute 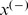, 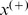 and *C* using positives and non-positives from the remaining 1/3 of the nodes in the training set.

## Results

### A Biological Example

Figure 1 illustrates the use of 3Prop on two different patterns of node label distribution in two types of biological networks. In these networks, the nodes represent proteins or genes and the task is to label nodes with the functions of their corresponding proteins. In the network shown in Figure 1A, the links connect proteins that physically interact with one another in the yeast cell and the positives proteins involved in “Meiotic Cell Cycle” according to Gene Ontology (GO) [28]. In Figure 1B, genes are linked if they have a synthetic lethal genetic interaction (*i.e.,* simultaneous mutation of both of their corresponding genes is fatal, but a mutation of only one is not). The positives in this example are genes involved in “Transcriptional Initiation”. In Figure 1A, nodes with the same label are assortatively mixed and in Figure 1B, positive nodes rarely link to each other but often share neighbors. These differences in network characteristics are typical of physical and genetic interaction networks [20], [29], [30]. GLP based algorithms do well in predicting node labels for 1A (performance illustrated in Figure 1M), but completely fail to identify the node labeling pattern presented in 1B (performance illustrated in Figure 1N). On this genetic interaction network example, GLP achieves an area under the ROC curve (AUROC) of 0.2 which is much worse than random performance–the low performance of GLP can be attributed to its fixed assumption that nodes connected by shorter paths are more likely to share labels.

The other panels in Figure 1, namely 1E-1L, illustrate the 3Prop algorithm; the random walk probabilities of length up to three along with the final 3Prop scores. For the physical interaction network, length two and three random walk probabilities are much better indicators of a positive label than the length one probabilities. For the genetic interaction network, the best single indicator of being a positive is to have a relatively low length three random walk probability. 3Prop detects these trends and assigns weights accordingly (see Table 1 for the weights). The resulting node scores for symmetric 3Prop (Figures 1K and 1L) for both labeling problems perfectly distinguish the true positives from the non-positives, resulting in an AUROC of 1 (asymmetric 3Prop achieves an AUROC of 0.83 on example 1B and an AUROC of 1 on example 1A).

**Table 1:** 3Prop coefficients assigned to walks of length one, two, and three.

experiment	1*^st^* step	2*^nd^* step	3*^rd^* step
Fig. 1A (PI)	0.022	0.68	0.29
Fig. 1B (GI)	−0.11	−0.22	−0.66
Caltech	0.072	0.45	−0.477
Princeton	0.063	0.45	−0.48
Georgetown	0.054	0.46	−0.48
Oklahoma	0.0081	0.51	−0.48
UNC	0.022	0.49	−0.48

### Experimental Performance of 3Prop

In our experiments we use a diverse collection of networks including: two types of molecular networks, protein-protein interaction (PI) and genetic interaction (GI) (downloaded from BioGRID [31]); five social networks representing Facebook friendship relationships between students at various universities [32]; a blog network capturing hyperlinks between political opinion blogs [33], and a patent-citation network where patents are linked whenever one cites the other [34] (see Table 2). We consider all edges as undirected. These networks vary in size from 750 nodes to 3 million nodes. We consider categories of biological function as labels for the PI and GI networks (we use 47 GO categories from GO fringe [35] that have between 30–300 annotations), gender as labels for the social networks, political view (liberal and conservative) as labels for the blog network, and assigned patent categories as labels for the patent-citation network (we only use patent categories that have more than 100 patents assigned to them). The chosen set of networks represents a broad variety of patterns in the proportions of nodes that are initially labeled with various categories. For example, about 40% of nodes in Facebook networks are initially labeled as male; in contrast, only 0.0002% of the nodes in the patent network are initially labeled with the category “Wheelwright Machines”.

**Table 2:** Networks used in this study.

Dataset	nodes	edges	average shortest distance	diameter	labels
Protein Interaction	5,405	414,242	2.5	7	47 protein functions
Negative Genetic Interaction	4,563	152,188	2.8	6	47 protein functions
Facebook^1^ (Caltech)	769	33,312	2.3	6	gender
Facebook^2^ (Georgetown)	9,414	851,276	2.7	11	gender
Facebook^3^ (Princeton)	6,596	586,640	2.7	9	gender
Facebook^4^ (Oklahoma)	17,425	1,785,056	2.7	9	gender
Facebook^5^ (UNC)	18,163	1,533,600	2.8	7	gender
Political Blogs	1,224	33,433	2.7	8	liberal or conservative
Patent Citation	3,774,768	33,037,894	8.5	23	381 patent categories

We compare the performance of symmetric 3Prop with that of symmetric GLP (SLP) which has been shown to perform well in gene function prediction problems [36]. We report the performance of SLP and 3Prop according to 3-fold cross-validation, where we determine the parameter settings using a further 2-fold cross-validation on the training fold.

We evaluate GLP and 3Prop using two standard measures: area under the ROC curve (AUROC) and average precision (AUP).The ROC curve is a graphical plot of recall (number of true positives divided by the total number of positives) as a function of false positive rate (number of false positives divided by the total number of negatives) for a binary classifier as we vary the discrimination threshold. The area under this curve (AUROC) can achieve a maximum value of 1 and a minimum of 0; a random classifier will result in AUROC of 0.5. AUROC can also be interpreted as the probability that a randomly chosen positive is assigned a discriminant score that is higher than a randomly chosen negative example. Precision at a given recall is defined as the fraction of predictions that are true positives and is given by TP/(TP+FP) where TP is the number of true positives and FP is the number of false positives at the given recall rate. A classifier that performs better in terms of AUROC is not guaranteed to perform better in terms of average precision, or vice versa. In general, average precision is a more suitable measure where there are many more non-positive compared to positive examples [37].

**Figure 2:**
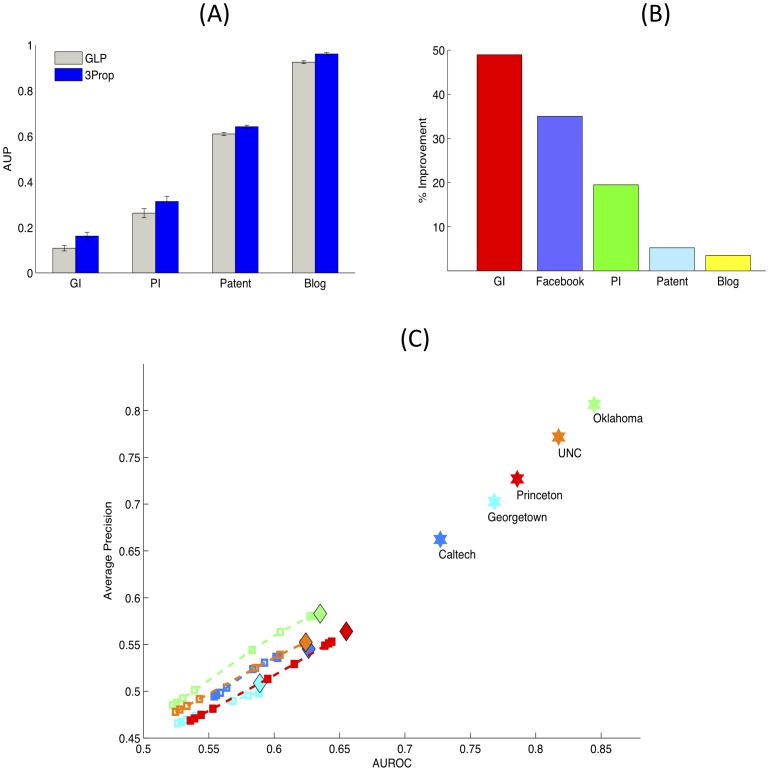
**Predictive accuracy of 3Prop.** (A) Average precision of SLP and 3Prop in prediction node labels using PI, GI, Facebook, patent, and blogs networks. (B) Percent improvement of symmetric 3Prop over symmetric GLP in average precision on the various networks. (C) Performance of 3Prop compared to GLP in area under the ROC curve (AUROC) (x-axis) and average precision (y-axis) in predicting gender from Facebook. Performance of GLP is shown for the range of settings of the parameter 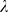 (lines). Stars indicate the performance of 3Prop, diamonds indicate the performance of 2Prop.

Figure 2 compares the performance of 3Prop to that of SLP. Figure 2A and B show the average precision and relative improvement in average precision on held-out data (using 3-fold cross-validation), respectively. As shown in Figure 2B, predicting cellular function annotations using the GI network in yeast (47 labeling tasks) where 3Prop results in 49% improvement; predicting the same annotations using the PI network in yeast where 3Prop results in 19% improvement; on the five Facebook networks, 3Prop results in an average improvement of 34%. GLP already performs well on the patent and blog networks, and using 3Prop results in more modest improvements of 5% and 3%, respectively. Figure 2B compares the performance of 3Prop and GLP in predicting gender from all five Facebook networks. Because approximately half of the nodes are positive examples in the gender prediction task, in addition to mean precision, we also show area under the ROC curve (AUC), which is suitable for evaluating balanced problems where the number of positives is similar to the number of negatives. This figure also shows the achievable range of performance of GLP for all settings of the free parameter 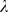. The performance of GLP is rather poor: on average about 40% of highly ranked nodes will have the opposite gender as the one predicted. As shown, the improvement of 3Prop over GLP is consistent across all five Facebook networks.

#### Why random walks of length three?

**Figure 3:**
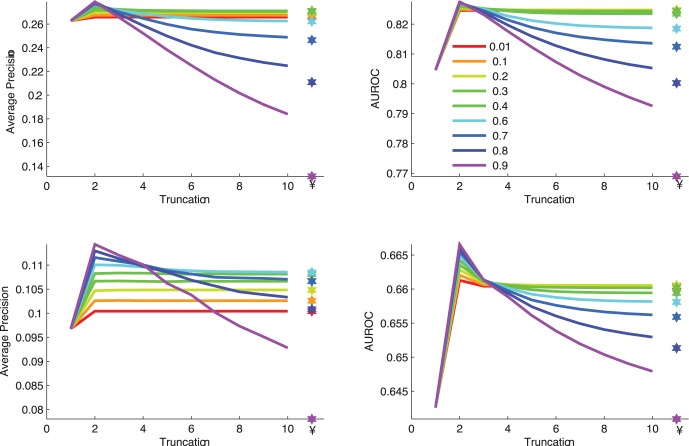
**The performance of truncated GLP with truncation level 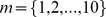 on (top) PI and (bottom) GI networks.** The different colored lines show the performance for varying values of the parameter 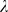. Stars show the performance of the exact solution to GLP.

**Figure 4:**
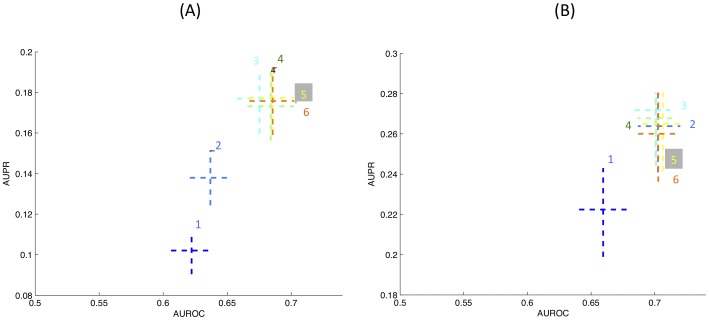
Average precision (AUPR) and area under the roc curve (AUROC) in predicting protein function from (A) GI and (B) PI networks, with 1Prop, 2Prop, 3Prop, 4Prop, 5Prop, and 6Prop. Here, we learn the random walk weights using the LDA algorithm (as in 3Prop). These plots show that considering random walks of length longer than 3 is unnecessary for accurate prediction of protein function from PI and GI networks.

As we have described, 3Prop only assigns non-zero weights to random walk probabilities that are shorter or equal to three. The choice of three is motivated by our observations about the performance of GLP with increasing random walk lengths, and average shortest node distances in several real-world networks (Table 2). For example, Figure 3 shows the performance of GLP with increasing random walk lengths on the two molecular interaction networks (PI and GI). As shown, the performance of GLP peaks with increasing random walk length up to three. The decrease in performance of GLP for 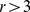, for some settings of 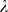, may be partially explained by the fast *convergence rate* of random walks on real-world networks (see the Discussion Section). In addition, we also observed that versions of 3Prop that consider longer random walks than three do not result in significant performance improvements. In particular, the area under the precision-recall curve of NProp peaks at 3Prop though there are some small gains in area under the ROC curve for 4, 5, and 6Prop (Fig. 4). This may reflect the greater ability provided by NProp for 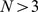 to distinguish nodes 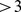 hops away from a positive but adding the additional parameters does decrease the average precision in predicting positive examples.

#### Interpreting 3Prop weights

**Figure 5:**
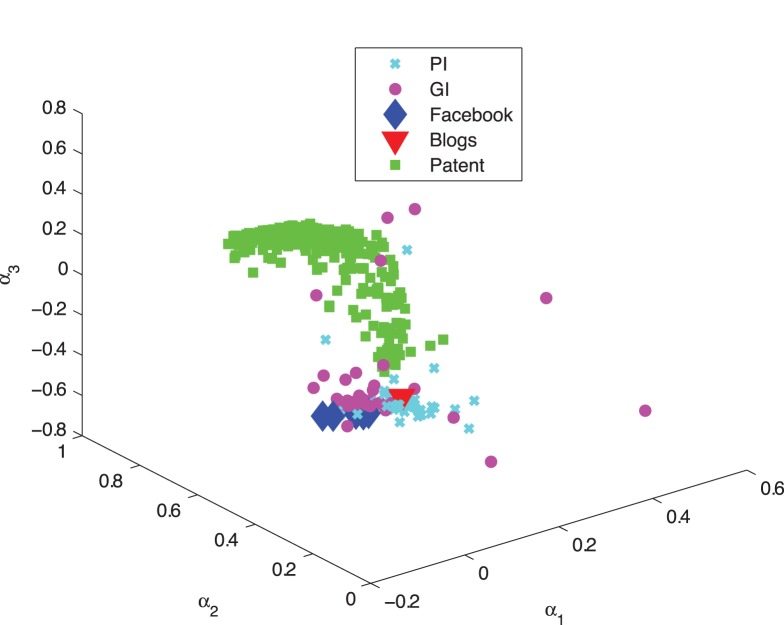
Weighting coefficients for walks of length one to three (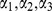) for the PI, GI, Blogs, Patent, and Facebook networks.

For all networks, except the patent network, weights assigned by 3Prop are similar based on the task or the network type (Figure 5). For example, in all the Facebook networks, 3Prop assigns a large positive weight to walks of length two, a large negative weight to those of length three and a negligible weight to walks of length one (Table 1). This surprising classification scheme assign a low weight to the gender of immediate friends but relies heavily on the gender of friends of friends of friends but is nonetheless much more accurate than any alternative; considering the three types of random walk probabilities separately result in poor performance (Table 3), as does a version of 3Prop for which the weight of random walks of length three is forced to be zero (*i.e.,* “2Prop”) (Figure 2). Note that if two nodes are connected by a random walk of length one, they are also connected by a random walk of length three. The good performance of 3Prop on this example then may be attributed to its capacity to exploit “double-counting”.

**Table 3:** Predicting gender from Facebook while using walks of length one, two, and three, individually.

network	1*^st^* step	2*^nd^* step	3*^rd^* step
Caltech	0.66	0.67	0.63
Georgetown	0.59	0.60	0.56
Oklahoma	0.67	0.65	0.61
Princeton	0.69	0.65	0.61
UNC	0.6394	0.61	0.57

The performance is shown in terms of AUROC.

**Figure 6:**
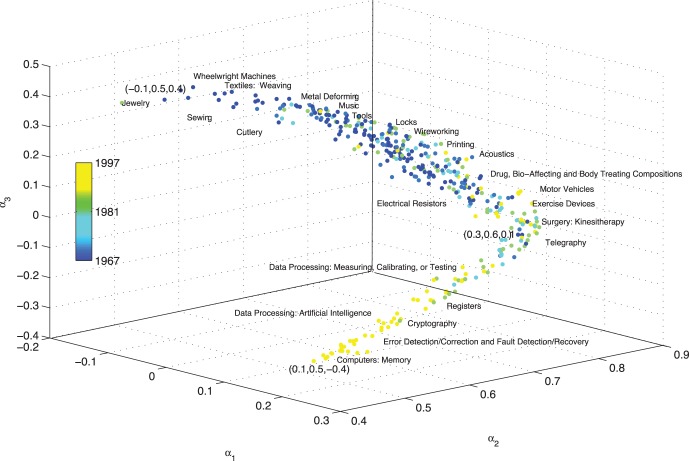
The coefficients of 3Prop assigned to 381 patent categories; each dot in the 3D space is defined by the 3Prop weights assigned to a particular patent category. The colors depict the age of the patent (when the patent was assigned).

In contrast, the 3Prop weights for the different patent categories vary considerably (Figure 6). However, these weights all lie on a curved line in 3-D space (Figure 6), and the location of weights on this curve reflects the average age of the patents within each category. Because patents can only cite older patents, this may reflect a structural evolution of the node distribution patterns in this network.

## Discussion

Despite its limitations, label propagation has become the algorithm of choice for many node labeling problems. It is easy to implement, resists overfitting because it has only a single free parameter but it nonetheless performs as well as or better than much more complex algorithms on benchmark problems [36], [38], [39]. Also, unlike more complex methods, such as multiple kernel learning with random walk kernels (*e.g.,* [40], [41]), it scales to large network-based classification problems.

3Prop retains all of the advantages of GLP but is faster and more accurate. If provided with 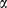, 3Prop’s node scores can be calculated exactly using three matrix-vector products, whereas GLP often requires many more iterations [22]. Computing 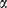 requires only as much time as computing the node scores. 3Prop has less than three free parameters, so only a small number of positive examples are required for training. Also, in some cases, one can use pre-defined weights learning for other labeling tasks on the same network.

Surprisingly, we have found that for many networks, the third iteration of label propagation receives a negative weight. Assigning these negative weights gives 3Prop the flexibility to exploit “over-counting” of random walks in real-world networks, where short random walk probabilities could have a large contribution to longer random walk probabilities.

3Prop only considers random walks of length up to three; a natural question is, “Why three and not more (or fewer)? ”. For example, even in assortatively mixed networks, having many paths of length two between two nodes is evidence that they are members of the same network module or “community” [42], and nodes in the same community often share labels. Furthermore, in some cases, higher order statistics of networks, expressed in terms of counting paths of length 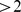, also contain some topological information helpful for predicting node label (*e.g.,* [43]). However, we have observed that for many real-world networks, assigning non-zero weights to random walk probabilities for 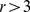 is unnecessary and may be counter-productive (*e.g.,* Figure 3). This may reflect the fact that the small average shortest path distances, which were less than three, in most of the networks that we considered, and networks with longer average shortest path distances may require more propagation steps. However, it could also reflect the fast *convergence rate* of random walks on real-world networks. In particular, all non-bipartite, connected networks have an associated *stationary distribution* over the nodes, 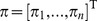, defined by 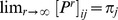, where 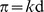, 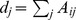 and 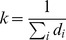. [44]. In other words, after a sufficiently long random walk, all information about the starting point of the walk is lost. So for sufficiently large *r*, the rows of 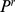 become nearly identical, and at this point, regardless of y, 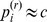 for some constant *c*. As such, once convergence is reached, adding 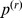 to the node scores does not change their relative rankings. Note that for SLP, assigning non-negligible, non-zero positive weight to longer random walks can decrease accuracy because as 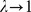, the total weight assigned to the values of *r* for the constant values of 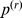 becomes large and as such, 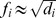. This effect may explain recent observations that node rankings based on GLP node scores and those based on weighted degree are very similar [45]. In most networks that we have examined, the random walk probabilities for 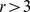 are already near their stationary distribution (see Figures S1 and S2), so considering these probabilities provides no additional information about node labeling. In summary, only short paths between nodes carry information about node labels because random walks in real-world networks converge quickly to the stationary distribution. We expect this to also be true for many other real-world networks because many of the topology properties shared by these networks–including small average shortest path distance between nodes (see Table 2) [46] or high betweenness centrality of hubs in the networks [47]–are properties that lead to fast convergence of random walks.

## Supporting Information

Figure S1Total variation distance between random walks of increasing length as a function of walk length *r* for the five Facebook networks and two molecular networks. Each grey line was generated by starting a random walk from a random node *i* and assessing the total variation distance between the distribution 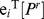 and 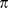, where 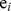 is a vector of 0 s, except for one 1 at position *i*. There are 100 grey lines, corresponding to 100 random selections of *i*. The red line shows the median. To obtain the convergence, we only consider the largest connected component for each network.(TIF)

Figure S2 Total variation distance between random walks of increasing length as a function of walk length *r* in the Blogs network.
(TIF)

Text S1 Supplementary methods.
(PDF)
