# Supplementary figures and images for: Labeling Nodes Using Three Degrees of Propagation
Source: PLoS One. 2012 Dec 28;7(12):e51947. doi: 10.1371/journal.pone.0051947 (PMC3532359; doi:10.1371/journal.pone.0051947)

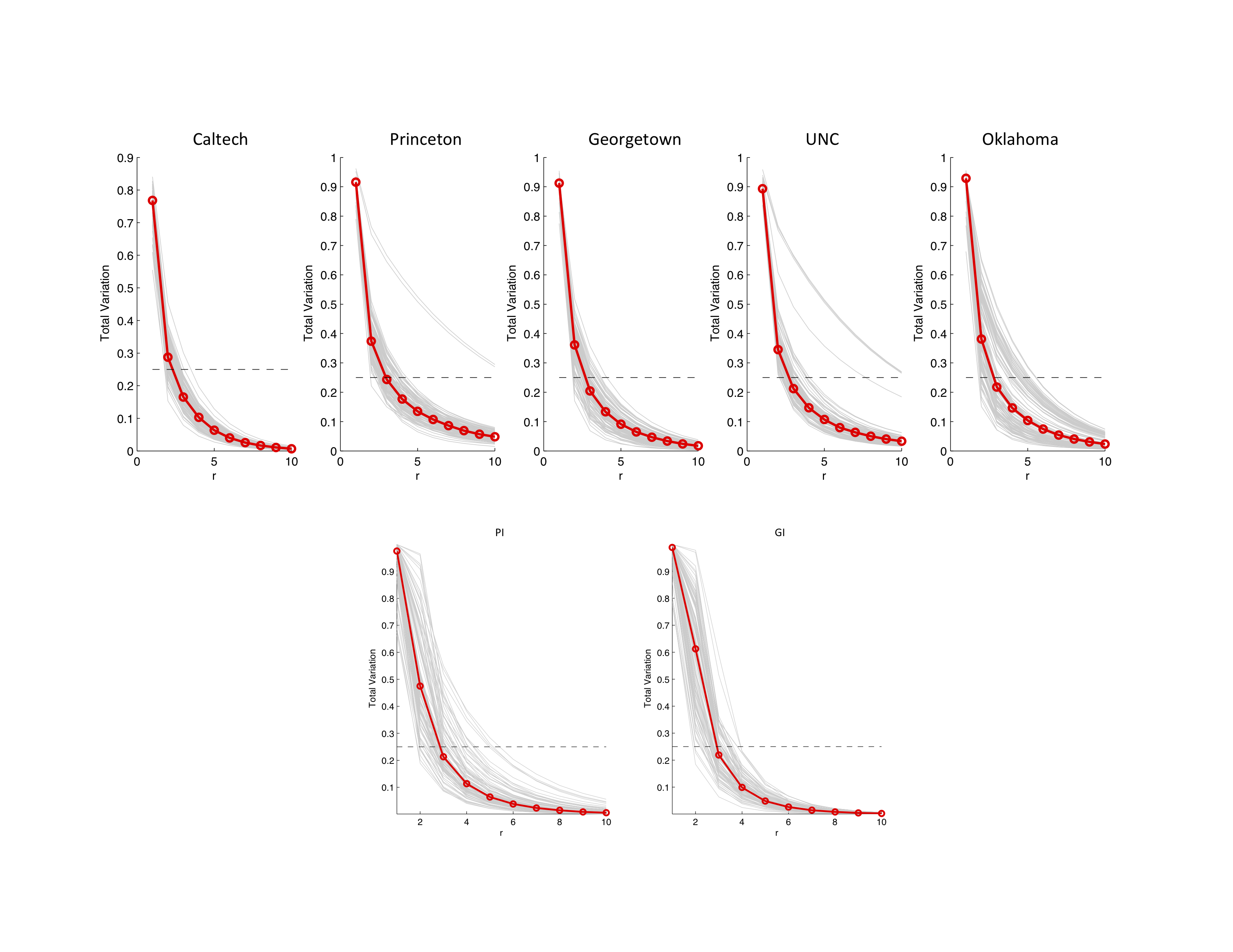

Supplement: Figure S1 — Total variation distance between random walks of increasing length as a function of walk length r for the five Facebook networks and two molecular networks. Each grey line was generated by starting a random walk from a random node i and assessing the total variation distance between the distribution and , where is a vector of 0 s, except for one 1 at position i. There are 100 grey lines, corresponding to 100 random selections of i. The red line shows the median. To obtain the convergence, we only consider the largest connected component for each network. (TIF) [file pone.0051947.s001.tif]

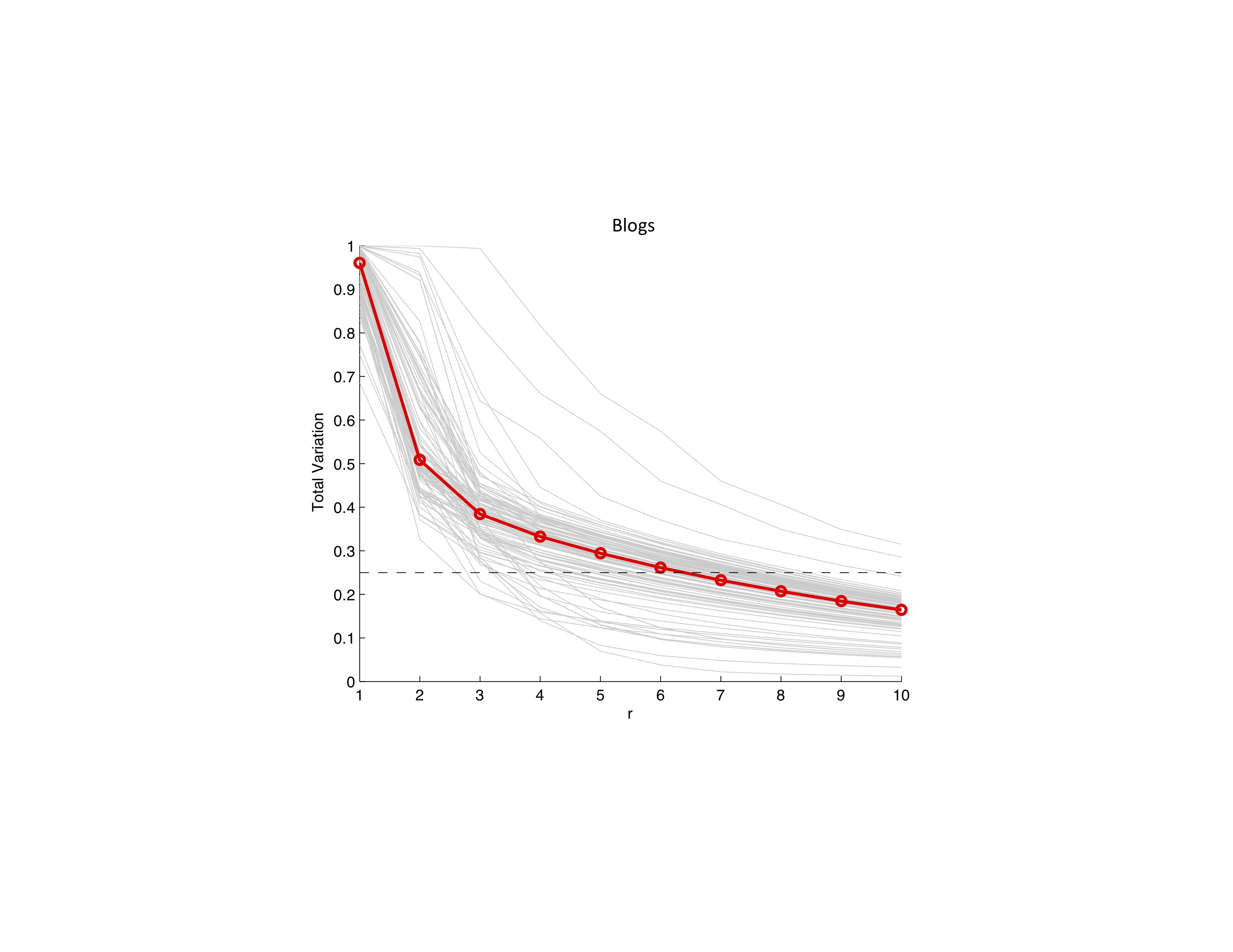

Supplement: Figure S2 — Total variation distance between random walks of increasing length as a function of walk length r in the Blogs network. (TIF) [file pone.0051947.s002.tif]
