# Supplementary material for: Labeling Nodes Using Three Degrees of Propagation
Source: PLoS One. 2012 Dec 28;7(12):e51947. doi: 10.1371/journal.pone.0051947 (PMC3532359; doi:10.1371/journal.pone.0051947)
Supplement: Text S1 — Supplementary methods. (PDF) [file pone.0051947.s003.pdf]

Supplement

GLP and Gaussian Random Fields, and other related approaches We can also derive the solution to the label propagation algorithm as inference in Gaussian Random fields. In particular, for a probabilistic formulation in the framework of inference on a Gaussian Random Field [5], the goal is to find the most likely assignment to the vector $\mathbf{f}$, given a likelihood term $p(\mathbf{y}|\mathbf{f})$ and a prior $p(\mathbf{f})$, both of which are Gaussians:

$$\begin{aligned}
\mathbf{f}^{(*)} &= \underset{\mathbf{f}}{\operatorname{argmax}}\, p(\mathbf{f}|\mathbf{y})p(\mathbf{f}) \\
&= \underset{\mathbf{f}}{\operatorname{argmax}}\, N(\mathbf{f}|\mathbf{y}, \sigma_o I)N(\mathbf{f}|\mathbf{0}, \sigma_p (I-S)^{-1}) \\
&= \underset{\mathbf{f}}{\operatorname{argmax}}\, N(\mathbf{f}|(1-\lambda)(I-\lambda S)^{-1}\mathbf{y}, (1-\lambda)(I-\lambda S)^{-1}) \\
&= (1-\lambda)(I-\lambda S)^{-1}\mathbf{y}\ , \quad 0 < \lambda = \frac{\sigma_p}{\sigma_o + \sigma_p} < 1
\end{aligned}$$

where $N(\mu, \mathbf{\Sigma})$ is the Gaussian distribution with mean μ and covariance matrix Σ, σ_o and σ_p are model parameters that balance the strength of the prior $p(\mathbf{f})$ with the fit of the model $p(\mathbf{y}|\mathbf{f})$. The last equality is derived by using the fact that the mode of the Gaussian distribution coincides with its mean. In the above formulation, we use the symmetrically normalized matrix S, as the covariance matrix must be symmetric. Again, note that $0 < \lambda < 1$ since $\sigma_o > 0$ and $\sigma_p > 0$.

Equivalently, we can derive the solution to the label propagation algorithm by optimizing an objective function comprised of a squared loss and a regularization term, where the regularization term encourages smoothness of the final scores over the *graph Laplacian* (*e.g.,* [6, 8]) (corresponding to optimizing the log of the above likelihood):

$$\mathbf{f}^{(*)} = \underset{\mathbf{f}}{\operatorname{argmin}}\, \tfrac{1}{\sigma_o}(\mathbf{f}-\mathbf{y})^{\mathsf{T}}(\mathbf{f}-\mathbf{y}) + \tfrac{1}{\sigma_p}\mathbf{f}^{\mathsf{T}}L\mathbf{f}$$

where L is the graph Laplacian ($L = D - A$, where D is the diagonal row sum matrix of A), or the normalized graph Laplacian $L = D^{-1/2}(D-A)D^{-1/2} = I - S$. Taking the derivative of the above and setting it to zero, we arrive at the label propagation solution: *i.e.,* $\mathbf{f} = (1-\lambda)(I-\lambda S)^{-1}\mathbf{y}$. Other related approaches, such as

the work of [6] and Iterated Laplacian [9], replace S with more general kernel matrices.

Other related algorithms include the diffusion-based label propagation algorithms described by [10, 8], RankProp [7] and PageRank [2]. The Rankprop algorithm [7] uses the asymmetrically normalized matrix P, whereas the personalized PageRank [2] algorithm first normalizes the label vector: $\tilde{\mathbf{y}} = \mathbf{y}/\sum_i y_i$, and then obtains the final scores as $(1-\lambda)\sum_{r=0}^{\infty}(\lambda P)^r\tilde{\mathbf{y}}$.

Several heuristic methods have also been derived which are related to a truncated version of GLP. For example, the BioPIXIE algorithm [3], only uses walks of length one and two (truncates the sum at $r = 2$), and P^2 only includes walks that go through the top m genes with highest direct neighbor scores. Nabieva and colleagues [4] also proposed a variation of label propagation called *FunctionalFlow*. Their approach does not explicitly set a decay parameter λ or down-weight the influence of hubs by normalization: these criteria are implicitly enforced by always propagating to shortest-distance neighbors first and subtracting *out-flow* from *in-flow*.

Random Walks and Convergence to Stationary Distribution

All sufficiently long random walks defined on a connected, aperiodic (or, equivalently, *ergodic*) network converge to an stationary distribution π[1]. At convergence, we have:

$$\lim_{r\to\infty} P^r = \mathbf{1}\pi^{\mathsf{T}} \tag{1}$$

where π and $\mathbf{1}$ are vectors of dimension $n \times 1$. In other words, for large r, P^r converges to a matrix with identical rows π^{T}, where $\pi = k\mathbf{d}$, $d_i = \sum_i W_{ij}$ and $k = \frac{1}{\sum_i d_i}$. Below, we describe how to calculate the number of steps required for the convergence of P^r.

At convergence, for any positive vector $\mathbf{v}$ with $\sum_i v_i = 1$ we have that $\lim_{r\to\infty} \mathbf{v}^{\mathsf{T}} P^r = \pi^{\mathsf{T}}$. The *mixing time* of a random walk quantifies how large r should be for $\mathbf{v}^{\mathsf{T}} P^r$ to approximately converge to π^{T}. Formally, mixing time is defined as the smallest r such that: $||\mathbf{v}^{\mathsf{T}} P^r - \pi^{\mathsf{T}}|| < \epsilon$ where $||.||$ denotes the chosen measure of the distance [1]. A standard distance metric for measuring convergence is the *total variation distance* defined as half of the ℓ_1-norm: $TV = \frac{1}{2}\sum_i \left|[\mathbf{v}^{\mathsf{T}} P^r]_i - \pi_i\right|$ and by convention $\epsilon = \frac{1}{4}$. Thus, we define the mixing time of the Markov transition matrix P as the smallest r such that the total variation distance between $\mathbf{v}^{\mathsf{T}} P^r$, for a randomly chosen vector $\mathbf{v}$ with $\sum_i v_i = 1$, and π is less than $\frac{1}{4}$:

$$\frac{1}{2}(\sum_i \left|[\mathbf{v}^{\mathsf{T}} P^r]_i - \pi_i\right|) < \frac{1}{4}. \tag{2}$$

Figures S1 and S2 show the convergence for the five Facebook networks, and the protein and genetic interaction networks. As shown, Facebook, PI and GI networks have median mixing times that are less than 3. This short mixing time explains the negative coefficients assigned to walks of length three with 3Prop. The Blogs network has a longer mixing time (around 6). One explanation for the longer mixing time of the Blogs network is that this network consists of two distinct clusters of nodes (the liberal cluster and the conservative cluster). There are an order of magnitude more edges within each cluster (the liberal cluster has a density of 0.038 and the conservative cluster has a density of 0.042) than between clusters (a density of 0.004), and this type of almost "bi-partite" structure may explain the increased mixing time of blogs network.

The convergence of random walks on the patent-citation network was longer than the other networks ($r \approx 12$), this is probably because the patent-citation network is much sparser than the other networks (density of 1.8×10^{-6}). Nonetheless, we found that 3Prop performed as well as GLP on this network.

The convergence of the random walk matrix P has practical implications for GLP algorithms. In particular, in the case of asymmetric GLP, after convergence of random walks, later iterations of label propagation simply add a constant to each node score. In contrast, in symmetric GLP, later iterations change the node scores by a none-label-specific score that depends on the square root of the node degrees. Thus, in the case of symmetric GLP, we note that if λ does not decay quickly enough, later iterations of GLP in fact can degrade the performance (as in the case of Figure 3).

References

[1] F.R. Chung. *Spectral Graph Theory.* CBMS Regional Conference Series in Mathematics. American Mathematical Society, 1997.

[2] T. Haveliwala. Topic-sensitive pagerank. *Proceedings of the Eleventh International World Wide Web Conference,*, 2002.

[3] C.L. Myers, D. Robson, A. Wible, M. Hibbs, C. Chiriac, C.L. Theesfeld, K. Dolinski, and O.G. Troyanskaya. Discovery of biological networks from diverse functional genomic data. *Genome Biology*, 6:R114, 2005.

[4] E. Nabieva, K. Jim, A. Agarwal, B. Chazelle, and M. Singh. Whole-proteome prediction of protein function via graph-theoretic analysis of interaction maps. *Bioinformatics*, 2(Suppl. 1), 2005.

[5] H. Rue and L. Held. *Gaussian Markov Random Fields: Theory and Applications*, volume 104 of *Monographs on Statistics and Applied Probability.* Chapman & Hall, London, 2005.

[6] A. Smola and R. Kondor. Kernels and regularization on graphs. *Conference on Learning Theory (COLT)*, 2003.

[7] J. Weston, A. Elisseeff, D. Zhou, C. Leslie, and W.S. Noble. Protein ranking: From local to global structure in the protein similarity network. *PNAS*, 101:6559–6563, 2004.

[8] D. Zhou, O. Bousquet, J. Weston, and B. Scholkopf. Learning with local and global consistency. *Advances in Neural Information Processing Systems*, 16:321–328, 2004.

[9] X. Zhou and M. Belkin. Semi-supervised learning by higher order regularization. *International Conference on Artificial Intelligence and Statistics (AISTATS)*, 2011.

[10] X. Zhu, Z. Ghahramani, and J. Lafferty. Semi-supervised learning using gaussian fields and harmonic functions. *Proceedings of International Conference on Machine Learning*, pages 912–919, 2003.
